# Association of *STAT3* Common Variations with Obesity and Hypertriglyceridemia: Protective and Contributive Effects

**DOI:** 10.3390/ijms150712258

**Published:** 2014-07-10

**Authors:** Zuliang Ma, Guanghai Wang, Xuejiao Chen, Zejin Ou, Fei Zou

**Affiliations:** 1 Department of Occupational Health and Occupational Medicine, School of Public Health and Tropical Medicine, Southern Medical University, Guangzhou 510515, China; E-Mails: mazulianghn@gmail.com (Z.M.); gongyuan1973@gmail.com (G.W.); chenxuejiaosx@gmail.com (X.C.); 2 Health Management Center, Nanfang Hospital, Southern Medical University, Guangzhou 510515, China; E-Mail: ouzejingd@gmail.com

**Keywords:** obesity, body-mass index, hypertriglyceridemia, genetic susceptibility, signal transducer and activator of transcription 3

## Abstract

Signal transducer and activator of transcription 3 (STAT3) plays an important role in energy metabolism. Here we explore whether *STAT3* common variations influence risks of obesity and other metabolic disorders in a Chinese Han population. Two tagging single nucleotide polymorphisms (tagSNPs), rs1053005 and rs957970, were used to capture the common variations of *STAT3*. Relationships between genotypes and obesity, body mass index, plasma triglyceride and other metabolic diseases related parameters were analyzed for association study in 1742 subjects. Generalized linear model and logistic regression model were used for quantitative data analysis and case-control study, respectively. rs1053005 was significantly associated with body mass index and waist circumference (*p* = 0.013 and *p* = 0.02, respectively). rs957970 was significantly associated with plasma level of triglyceride (*p* = 0.007). GG genotype at rs1053005 had lower risks of both general obesity and central obesity (OR = 0.40, *p* = 0.034; OR = 0.42, *p* = 0.007, respectively) compared with AA genotype. CT genotype at rs957970 had a higher risk of hypertriglyceridemia (OR = 1.43, *p* = 0.015) compared with TT genotype. Neither of the two SNPs was associated with othermetabolic diseases related parameters. Our observations indicated that common variations of *STAT3* could significantly affect the risk of obesity and hypertriglyceridemia in Chinese Han population.

## 1. Introduction

Obesity is a widespread health problem nowadays. It is a leading risk factor for several serious diseases such as type 2 diabetes, cardiovascular disease, and certain cancers [1]. Signal transducer and activator of transcription 3 (STAT3), is a ubiquitous cytoplasmic protein expressed in multiple metabolic tissue. This protein is a member of the STAT protein family, which, in response to a variety of cytokines and growth factors, can be activated through phosphorylation and then translocate to the cell nucleus and act as transcription factors there [2]. It has been well established that STAT3 activation in hypothalamus plays a critical role in the leptin-mediated regulation of energy metabolism [3]. The long form of the leptin receptor–STAT3 signal is proven to be central to the regulation of food intake and energy expenditure by leptin [4]. Deletion of STAT3 in hypothalamus has been demonstrated to interfere with normal body weight homeostasis and glucose metabolism [5]. Experiments using knockout mouse model showed that the disruption of neural STAT3 causes apparent leptin-resistant conditions such as obesity, diabetes and thermal dysregulation [6]. Besides, mice with a pancreatic beta-cell-specific disruption of the *STAT3* gene exhibited an increase in appetite and obesity, and partial leptin resistance and glucose intolerance [7]. Loss of STAT3 in mature adipocytes in mice displayed increased adiposity and adipocyte hypertrophy [8]. In addition, loss- and gain-of-function experiments in a mouse model showed that STAT3 played a crucial role in regulating the expression of gluconeogenic genes in the liver, and thus in normal glucose homeostasis, which means STAT3 is also indispensable for the function of insulin [9,10]. Interestingly, a recent study in a mouse model showed that STAT3 could regulate the differentiation of brown adipose tissues (BAT), which mainly act for burning energy [11].

Since STAT3 plays important roles in almost every aspect of energy metabolism, its variations might predispose the carriers to some risks of the metabolic diseases. To our knowledge, limited studies have been done so far to investigate this question. Four previous researches concerning that issue were all performed in populations of European ancestry, and had produced inconsistent results [12,13,14,15]. To investigate whether the polymorphisms of *STAT3* confer risks of metabolic disorders in Chinese population, we conducted this association study and found that the common variants of *STAT3* were significantly associated with obesity and hypertriglyceridemia in a Chinese Han population.

## 2. Results

The demographic and biochemical data of this study was summarized in Table 1. The data was organized in gender, age and clinical measurements by weight status. All of the participants were grouped into healthy weight, overweight, general obesity or central obesity according to the criterion described previously. The distribution of our participants among the four groups was significantly different between males and females (*p* < 0.005). The average of age and the clinical measurements were all significantly different among the four groups (*p* < 0.005 for all variables). Since age and gender influence weight status profoundly, the following analyses were all adjusted for these two confounding variables as appropriate. All of our subjects were genotyped successfully, and the genotypes of randomly select samples were totally confirmed by sequencing.

**Table 1:** Demographic and biochemical characteristics of study on association between *STAT3* and the risks of metabolic disorders.

Variable		All	Overweight	General Obesity	Central Obesity	Healthy Weight	*p* ^a^
Gender	Male (%)	702 (40.3) ^b^	291 (61.7)	88 (77.2)	140 (59.1)	227 (26.1)	<0.005
	Female (%)	1040 (59.7) ^b^	181 (38.3)	26 (22.8)	97 (40.9)	644 (73.9)	
Age (years)		37.40 ± 10.97 (18–85)	41.19 ± 10.82	43.63 ± 11.00	43.53 ± 11.17	34.67 ± 9.53	<0.005
BMI (kg/m^2^)		23.03 ± 3.36 (15.12–37.09)	25.69 ± 1.10	30.05 ± 2.15	27.45 ± 2.66	21.33 ± 1.50	<0.005
FG (mmol/L)		4.88 ± 0.79 (3.0–14.0)	5.05 ± 0.82	5.32 ± 1.11	5.20 ± 0.92	4.73 ± 0.54	<0.005
TG (mmol/L)		1.29 ± 1.09 (0.25–16.59)	1.72 ± 1.38	2.08 ± 1.50	1.92 ± 1.53	0.96 ± 0.69	<0.005
TC (mmol/L)		4.81 ± 0.96 (2.42–9.71)	5.09 ± 0.98	5.02 ± 1.04	5.03 ± 0.96	4.63 ± 0.88	<0.005
HDL (mmol/L)		1.76 ± 0.43 (0.73–3.42)	1.60 ± 0.38	1.43 ± 0.32	1.50 ± 0.33	1.88 ± 0.41	<0.005
LDL (mmol/L)		2.46 ± 0.72 (0.68–5.59)	2.71 ± 0.75	2.69 ± 0.73	2.66 ± 0.68	2.30 ± 0.64	<0.005
SBP (mm Hg)		114.91 ± 15.62 (78–190)	122.03 ± 15.40	127.78 ± 15.77	125.33 ± 15.03	110.57 ± 13.40	<0.005
DBP (mm Hg)		72.34 ± 10.16 (41–125)	76.33 ± 10.40	80.85 ± 10.52	78.68 ± 10.34	69.57 ± 8.62	<0.005
WC (cm)		75.60 ± 10.67 (47–116)	84.24 ± 6.69	94.99 ± 7.89	91.58 ± 6.89	70.81 ± 6.43	<0.005

BMI, body mass index; FG, fasting glucose; TG, triglyceride; TC, total cholesterol; HDL, high-density lipoprotein; LDL, low-density lipoprotein; SBP, systolic blood pressure; DBP, diastolic blood pressure; WC, waist circumference; ^a^ Comparison between overweight + general obesity and healthy weight, Chi-square test for difference of distribution of BMI status between male and female, Student’s *t* test for other variables; and ^b^ BMI of 96 males and 189 females was not measured due to personal reasons.

The polymorphism rs1053005 was significantly associated with BMI and waist circumference. Furthermore, BMI was inversely associated with the number of minor allele of rs1053005 (*p* = 0.013). Analysis with generalized linear model did not find significant associations between rs1053005 and any other clinical measurements including systolic blood pressure, diastolic blood pressure, fasting glucose, plasma triglyceride, total cholesterol, high density lipoprotein and low density lipoprotein. The polymorphism rs957970 was significantly associated with plasma level of triglyceride (*p* = 0.007), but was not associated with BMI or other measurements (Table 2).

**Table 2:** The associations between signal transducer and activator of transcription 3 (*STAT3*) polymorphisms and body mass index (BMI) or serum indices ^a^.

Variable	rs1053005			*p*	rs957970			*p*
	AA	AG	GG		TT	CT	CC	
BMI (kg/m^2^)	23.23 ± 3.54	22.88 ± 3.25	22.76 ± 2.91	0.013	23.10 ± 3.44	23.08 ± 3.35	22.71 ± 3.19	0.41
FG (mmol/L)	4.87 ± 0.78	4.89 ± 0.76	4.95 ± 0.92	0.62	4.86 ± 0.74	4.90 ± 0.77	4.90 ± 0.92	0.54
TG (mmol/L)	1.24 ± 0.87	1.33 ± 1.27	1.39 ± 1.17	0.25	1.20 ± 0.81	1.38 ± 1.30	1.26 ± 1.01	0.007
TC (mmol/L)	4.81 ± 0.94	4.81 ± 0.96	4.80 ± 1.04	0.91	4.82 ± 0.92	4.82 ± 0.98	4.77 ± 0.98	0.69
HDL (mmol/L)	1.77 ± 0.43	1.75 ± 0.42	1.72 ± 0.45	0.36	1.78 ± 0.43	1.74 ± 0.42	1.76 ± 0.44	0.46
LDL (mmol/L)	2.46 ± 0.72	2.45 ± 0.70	2.46 ± 0.76	0.95	2.47 ± 0.70	2.46 ± 0.72	2.42 ± 0.73	0.52
WC (cm)	76.20 ± 11.06	75.05 ± 10.40	75.23 ± 9.94	0.02	75.74 ± 10.70	75.76 ± 10.69	74.82 ± 10.58	0.90

BMI, body mass index; FG, fasting glucose; TG, triglyceride; TC, total cholesterol; HDL, high-density lipoprotein; LDL, low-density lipoprotein; WC, waist circumference and ^a^ adjusted for age and gender.

We further analyzed the association of the polymorphisms of *STAT3* with risk of metabolic diseases through case-control study. The distributions of genotypes for the investigated single nucleotide polymorphisms (SNPs) in control groups were consistent with Hardy–Weinberg equilibrium (*p* > 0.05). In terms of rs1053005, we found that, compared with AA genotype, carriers of G allele exhibited lower risks of both general obesity (OR = 0.47, 95% CI = 0.30–0.73, *p* = 0.001) and central obesity (OR = 0.69, 95% CI = 0.51–0.94, *p* = 0.019), which suggested a dominant protective effect of this A>G variation. The distributions of G and A alleles were significantly different between each of the two case groups and their corresponding control groups (22.8% *vs.* 33.1%, *p* = 0.002, and 27.0% *vs.* 32.9%, *p* = 0.012). In the locus of rs957970, we found that CT genotype had higher risk of hypertriglyceridemia, compared with TT genotype (OR = 1.43, 95% CI = 1.07–1.90, *p* = 0.015). (Table 3 and Table 4).

**Table 3:** Genotype frequencies of *STAT3* polymorphism rs1053005 and its association with the risks of metabolic disorders.

Variable	Genotype	Case	Control	OR Crude	95% CI	*p*	OR Adjusted	95% CI	*p* ^a^
		N (%)	N (%)						
Overweight	AA	217 (46.0)	391 (44.9)	1.00			1.00		
	AG	206 (43.6)	384 (44.1)	0.97	0.76–1.23	0.78	0.95	0.73–1.23	0.69
	GG	49 (10.4)	96 (11.0)	0.92	0.63–1.35	0.67	0.80	0.52–1.22	0.29
	Dominant			0.96	0.76–1.20	0.70	0.91	0.71–1.17	0.48
	A	640 (67.8)	1166 (66.9)	1.00					
	G	304 (32.2)	576 (33.1)	0.96	0.81–1.14	0.65			
General Obesity	AA	70 (61.4)	391 (44.9)	1.00			1.00		
	AG	36 (31.6)	384 (44.1)	0.52	0.34–0.80	0.003	0.49	0.30–0.78	0.003
	GG	8 (7.0)	96 (11.0)	0.47	0.22–1.00	0.05	0.40	0.17–0.93	0.034
	Dominant			0.51	0.34–0.76	0.001	0.47	0.30–0.73	0.001
	A	176 (77.2)	1166 (66.9)	1.00					
	G	52 (22.8)	576 (33.1)	0.60	0.43–0.84	0.002			
Hypertriglyceridemia	AA	155 (45.1)	611 (46.8)	1.00			1.00		
	AG	150 (43.6)	561 (42.9)	1.05	0.82–1.36	0.68	1.04	0.79–1.37	0.78
	GG	39 (11.3)	135 (10.3)	1.14	0.77–1.70	0.52	1.04	0.67–1.59	0.87
	Dominant			1.07	0.84–1.36	0.58	1.04	0.80–1.34	0.78
	A	460 (66.9)	1783 (68.2)	1.00					
	G	228 (33.1)	831 (31.8)	1.06	0.89–1.28	0.50			
Borderline-Hypercholesterolemia	AA	103 (45.8)	529 (46.4)	1.00			1.00		
	AG	104 (46.2)	490 (43.0)	1.09	0.81–1.47	0.57	1.08	0.80–1.46	0.63
	GG	18 (8.0)	120 (10.6)	0.77	0.45–1.32	0.34	0.73	0.42–1.27	0.27
	Dominant			1.03	0.77–1.37	0.86	1.01	0.76–1.36	0.93
	A	310 (68.9)	1548 (68.0)	1.00					
	G	140 (31.1)	730 (32.0)	0.96	0.77–1.20	0.70			
Hypercholesterolemia	AA	134 (46.7)	529 (46.4)	1.00			1.00		
	AG	117 (40.8)	490 (43.0)	0.94	0.72–1.24	0.68	0.91	0.68–1.21	0.50
	GG	36 (12.5)	120 (10.6)	1.18	0.78–1.80	0.43	1.10	0.70–1.71	0.68
	Dominant			0.99	0.76–1.28	0.94	0.95	0.72–1.24	0.69
	A	385 (67.1)	1548 (68.0)	1.00					
	G	189 (32.9)	730 (32.0)	1.04	0.85–1.27	0.69			
Central Obesity	AA	124 (52.3)	488 (45.0)	1.00			1.00		
	AG	98 (41.4)	479 (44.2)	0.81	0.60–1.08	0.15	0.77	0.56–1.05	0.10
	GG	15 (6.3)	118 (10.8)	0.50	0.28–0.89	0.018	0.42	0.23–0.79	0.007
	Dominant			0.75	0.56–0.99	0.04	0.69	0.51–0.94	0.019
	A	346 (73.0)	1455 (67.1)	1.00					
	G	128 (27.0)	715 (32.9)	0.75	0.60–0.95	0.01			

OR, odds ratio; 95% CI, 95% confidence interval; ^a^ Adjusted for age and gender.

**Table 4:** Genotype frequencies of *STAT3* polymorphism rs957970 and its association with the risks of metabolic disorders.

Variable	Genotype	Case	Control	OR Crude	95% CI	*p*	OR Adjusted	95% CI	*p* ^a^
		N (%)	N (%)						
Overweight	TT	166 (35.2)	335 (38.5)	1.00			1.00		
	CT	233 (49.4)	390 (44.8)	1.21	0.94–1.54	0.14	1.22	0.93–1.60	0.14
	CC	73 (15.4)	146 (16.7)	1.01	0.72–1.41	0.96	1.06	0.73–1.54	0.77
	Dominant			1.15	0.91–1.46	0.23	1.18	0.91–1.52	0.21
	T	565 (59.9)	1060 (60.8)						
	C	379 (40.1)	682 (39.2)	1.04	0.88–1.23	0.61			
General Obesity	TT	50 (43.9)	335 (38.5)	1.00			1.00		
	CT	50 (43.9)	390 (44.8)	0.86	0.57–1.31	0.48	0.84	0.53–1.33	0.46
	CC	14 (12.2)	146 (16.7)	0.64	0.34–1.20	0.16	0.74	0.37–1.45	0.38
	Dominant			0.80	0.54–1.19	0.27	0.81	0.52–1.25	0.34
	T	150 (65.8)	1060 (60.8)						
	C	78 (34.2)	682 (39.2)	0.81	0.60–1.09	0.15			
Hypertriglyceridemia	TT	109 (31.7)	504 (38.6)	1.00			1.00		
	CT	180 (52.3)	588 (45.0)	1.42	1.09–1.85	0.01	1.43	1.07–1.90	0.015
	CC	55 (16.0)	215 (16.4)	1.18	0.82–1.70	0.36	1.18	0.80–1.74	0.41
	Dominant			1.35	1.05–1.74	0.019	1.36	1.04–1.79	0.027
	T	398 (57.8)	1596 (61.1)						
	C	290 (42.2)	1018 (38.9)	1.14	0.96–1.36	0.13			
Borderline-Hypercholesterolemia	TT	92 (40.9)	418 (36.7)	1.00			1.00		
	CT	95 (42.2)	535 (47.0)	0.81	0.59–1.10	0.18	0.79	0.58–1.09	0.15
	CC	38 (16.9)	186 (16.3)	0.93	0.61–1.41	0.73	0.93	0.61–1.42	0.74
	Dominant			0.84	0.63–1.12	0.24	0.83	0.62–1.11	0.21
	T	279 (62.0)	1371 (60.2)						
	C	171 (38.0)	907 (39.8)	0.93	0.75–1.15	0.47			
Hypercholesterolemia	TT	103 (35.9)	418 (36.7)	1.00			1.00		
	CT	138 (48.4)	535 (47.0)	1.05	0.79–1.39	0.75	1.00	0.75–1.35	0.99
	CC	46 (16.0)	186 (16.3)	1.00	0.68–1.48	0.99	1.00	0.67–1.51	0.99
	Dominant			1.04	0.79–1.36	0.80	1.00	0.76–1.33	1.00
	T	344 (59.9)	1371 (60.2)						
	C	230 (40.1)	907 (39.8)	1.01	0.83–1.22	0.91			
Central Obesity	TT	93 (39.2)	406 (37.4)	1.00			1.00		
	CT	110 (46.4)	502 (46.3)	0.96	0.71–1.30	0.78	0.92	0.66–1.28	0.62
	CC	34 (14.4)	177 (16.3)	0.84	0.55–1.29	0.42	0.92	0.58–1.46	0.72
	Dominant			0.93	0.69–1.24	0.60	0.92	0.67–1.25	0.58
	T	296 (62.4)	1314 (60.6)						
	C	178 (37.6)	856 (39.4)	0.92	0.75–1.14	0.44			

OR, odds ratio; 95% CI, 95% confidence interval; ^a^ Adjusted for age and gender.

Analysis of the haplotypes composed of rs1053005 and rs957970 showed that, compared with the most common haplotype AT, both GT and GC haplotypes had a lower risk of general obesity (OR = 0.17, 95% CI: 0.04–0.69, *p* = 0.02; OR = 0.67, 95% CI: 0.48–0.94, *p* = 0.04, respectively), and GT haplotype also had a lower risk of central obesity (OR = 0.31, 95% CI: 0.14–0.71, *p* = 0.006), which suggested a potential epistatic effect of rs957970 on rs1053005 (Table 5).

**Table 5:** Estimated frequencies of haplotypes composed of rs1053005 and rs957970 and their association with metabolic disorders.

Variable	Haplotype	Case	Control	OR	95% CI	*p* ^a^
		NO. of Chromosomes (%)	NO. of Chromosomes (%)			
Overweight	AT	538 (57.0)	966 (55.5)	Base		
	GT	28 (2.9)	94 (5.4)	0.55	0.35–0.86	0.003
	AC	102 (10.8)	200 (11.5)	0.92	0.71–1.20	0.81
	GC	276 (29.3)	482 (27.7)	1.03	0.86–1.23	0.47
General Obesity	AT	148 (64.8)	966 (55.5)	Base		
	GT	2 (1.0)	94 (5.4)	0.17	0.04–0.69	0.02
	AC	28 (12.4)	200 (11.5)	0.93	0.61–1.43	0.59
	GC	50 (21.8)	482 (27.7)	0.67	0.48–0.94	0.04
Hypertriglyceridemia	AT	376 (54.7)	1474 (56.4)	Base		
	GT	22 (3.2)	122 (4.7)	0.73	0.45–1.18	0.15
	AC	84 (12.2)	310 (11.8)	1.06	0.82–1.39	0.68
	GC	206 (30.0)	708 (27.1)	1.14	0.94–1.38	0.18
Borderline-hypercholesterolemia	AT	258 (57.2)	1270 (55.8)	Base		
	GT	22 (4.8)	100 (4.4)	1.04	0.64–1.70	0.74
	AC	52 (11.7)	278 (12.2)	0.94	0.68–1.29	0.75
	GC	118 (26.3)	630 (27.6)	0.93	0.73–1.18	0.57
Hypercholesterolemia	AT	322 (56.0)	1270 (55.8)	Base		
	GT	22 (3.9)	100 (4.4)	0.90	0.56–1.44	0.72
	AC	64 (11.1)	278 (12.2)	0.91	0.68–1.23	0.50
	GC	166 (29.0)	630 (27.6)	1.05	0.85–1.29	0.57
Central Obesity	AT	290 (61.0)	1212 (55.9)	Base		
	GT	6 (1.5)	102 (4.7)	0.31	0.14–0.71	0.006
	AC	56 (12.0)	242 (11.2)	0.98	0.72–1.34	0.46
	GC	122 (25.6)	614 (28.3)	0.82	0.65–1.04	0.20

^a^ *p* was calculated with permutation test (1000 times).

## 3. Discussion

In this study, we explored the association between *STAT3* polymorphisms with obesity and other metabolic disorder related phenotypes in a Chinese Han population. We found associations between two tagSNPs with obesity or hypertriglyceridemia, but failed to find associations between any of them and other metabolic indices. Since several previous experimental studies have established the indispensable role of STAT3 in the regulation of body weight and energy metabolism, our findings are biologically plausible.

Animal researches reveal that STAT3 acts to fight against obesity through various distinctive pathways. Our observations of the protective effect of rs1053005 A>G variation suggested that carriers of G allele might have a higher expression or activity of STAT3. As to the function of STAT3 in the regulation of lipid metabolism, there are inconsistent observations. Though one report showed that over-expression of STAT3 in liver increased both the abundance of mRNA of lipogenic genes and the plasma level of triglyceride, most of other experiments indicated that STAT3 could significantly decrease the expression of lipogenic genes by inhibiting sterol regulatory element binding protein 1c (SREBP1c) and thus ameliorate the plasma level of triglyceride [16,17,18,19,20]. In the present study, we observed that the heterozygote CT for rs957970 increased the risk of hyperlipidemia by 1.43 times in comparison with the major TT homozygote, which suggested that C allele carriers for rs957970 might have lower expression of STAT3, in view of the major observations of the down-regulatory effects of STAT3 on lipogenic genes expression. What’s more, the up-regulatory effect of rs957970 C allele on the plasma level of triglyceride observed in our case-control study was in accordance with our quantitative analysis. But it is somewhat difficult to explain the relative smaller effects of the homozygous CC genotype, which was expected to have an increasing effect.

Linkage disequilibrium analysis showed that rs1053005 is in high linkage disequilibrium with the common SNPs located in the 14 kbps region of the 3' end of *STAT3* gene. As rs1053005 is sited in the 3' untranslated region, this variation may exert its effect by influencing micro-RNA targeting and thus affecting mRNA stability or translation efficiency of *STAT3* gene. Intriguingly, we found a putative target sequence for has-miR-1303 at locus of rs1053005 using the online microRNA target prediction tool RNA22 (version 1.0) [21]. Because the wild-type A allele of rs1053005 is complementary to the second nucleotide of the seed sequence of has-miR-1303, the variation from adenine to guanine may impair the perfect fitting between the seed of has-miR-1303 and its target, and thus hinder its down-regulatory effect, which may lead to higher expression level of STAT3 [22].

Though intronic variations might influence the expression of genes by affecting transcription efficiency or mRNA splicing, no solid experimental evidences support rs957970 functions through such mechanisms so far. The more likely explanation for the effect of this SNP would lie in other functional variants tagged by it. Because rs4796793 (C>G) in the putative promoter region (−1805 from the transcriptional start site) is in high linkage disequilibrium with rs957970 (*r*^2^ = 0.95), we supposed the former SNP was the exact causal variant for the effect of rs957970 and analyzed its function bioinformatically. Analysis with SNPInspector (version 2.4, Genomatix Inc., Munich, Germany) revealed that substitution of cytosine with guanine at rs4796793 could generated a transcription factor binding site for FOXD1, which have been shown to repress transcription of downstream genes in a tissue-specific manner [23,24]. It is possible that transcription of the mutant *STAT3* gene with the substitutive guanine at rs4796793 might be down-regulated by FOXD1, but further cellular and molecular experiments are required to testify this assumption.

There are hitherto four published reports investigating the association of *STAT3* polymorphisms with obesity and metabolic disorders, but the observations are inconsistent with each other. The earliest two researches were performed in female European of UK and male European of Buenos Aires, Argentina, respectively, and found no association between polymorphisms of *STAT3* and obesity [12,13]. But the following report by Catherine conducted in French showed that minor G allele of three tagSNPs (rs8069645, rs744166, and rs1053005) and major GG genotype of rs2293152 were all associated with increased risk of central obesity [14]. The recent published report by Li conducted in European Americans suggested that minor G allele of rs4796793 could decrease the risk of obesity [15]. As rs4796793 is in high linkage disequilibrium with rs8069645, the observation of Li implied a protective effect of minor G allele of rs8069645, which is contrary to the observation by Catherine [14]. Our study also support that polymorphisms of *STAT3* are associated with risk of obesity, but the protective effect of our investigated SNP rs1053005 is contrary to the findings by Catherine. Furthermore, our research suggests that rs4796793 might be associated with plasma level of triglyceride instead of obesity. The discrepancy among the above studies, we think, may be derived from difference in sample size, ethnic genetic background, lifestyle, dietary pattern, and gene-environment interactions.

The limitations of our study should be noted. First, the relatively limited sample size might lead to occurrence of statistical deviations by chance, and impair the statistical power of our study. Second, because of the effects of ethnic genetic background and gene-environment interaction, our observation might only make sense in Chinese population. Hence, cautions should be taken in interpreting our results, and association studies with larger samples in other populations are required to confirm the association between *STAT3* SNPs and obesity or hypertriglyceridemia. Analysis of STAT3 activity in tissues such as blood or fat with different genotypes would also be important to testify our observation, which remains to be done in our following research. Third, since the two tagSNPs we examined only capture the common SNPs in *STAT3* gene, we are not able to exclude the potential association between other rare variants of this gene and metabolic disorders related phenotypes. Though six tests were performed for each SNP in our study, an individual null hypothesis was tested for each association because of the independency of each kind of metabolic disorders in study. Since both of the two SNPs in our study were located in *STAT3*, the α value should be adjusted to be 0.05/2 = 0.025 to ensure that the association between *STAT3* gene and obesity or other metabolic disorders is certain at a 95% significance level. Our main conclusions on the association between *STAT3* polymorphisms and metabolic disorders remain statistically significant with the significance level of 0.025.

## 4. Experimental Section

### 4.1. Subjects

We recruited 1742 unrelated ethnic Chinese Han subjects including 871 health weights and 871 overweight or obesity. This sample size could provide more than 85% power to detect odds ratios of ≥1.5 for a disease with prevalence of 10% associated with disease allele frequency of 30%. All the subjects were recruited randomly from the individuals who got general health examinations in the Health Management Center, Nanfang Hospital, Guangzhou, China, from September 2009 to February 2011. Epidemical questionnaires were designed to collect data for age, gender, personal medical history, familial history, nutrition, and physical activity. All of the participants were confined to adults aged more than 18-year-old. Individuals who had familial history of metabolic diseases such as familial obesity, hypertension, dyslipidemia and hypercholesterolemia or were under anti-hyperlipidemia intervention were excluded. Individuals who did high intensity exercise regularly were also excluded. All participants were checked for body mass index (BMI, kg/m^2^), waist circumference (WC), serum lipids and fasting glucose (FG). All the measurements were executed by specialists in Nanfang Hospital, Guangzhou, China. Our study was approved by the institutional review board of Southern Medical University on 5 August 2009, and informed consents were obtained from each subject.

Metabolic disorders were diagnosed according to the updated National Cholesterol Education Program Adult Treatment Panel III criteria (NCEP-ATP III): hypertriglyceridemia:triglyceride (TG) ≥ 1.7 mmol/L; borderline hypercholesterolemia:total cholesterol (TC), 5.18–5.71 mmol/L; hypercholesterolemia:total cholesterol (TC) ≥ 5.72 mmol/L [25]. General obesity, overweight and health control for Chinese were defined as follow: BMI ≥ 28 kg/m^2^; BMI = 24–28 kg/m^2^; and BMI = 18.5–24 kg/m^2^, respectively [26]. Central obesity for Chinese was defined as follows: waist circumference ≥ 90/80 cm (men/women) [27].

### 4.2. Selection of Single Nucleotide Polymorphisms and Genotyping

Single nucleotide polymorphisms (SNPs) of *STAT3* gene were acquired from NCBI (build 36, dbSNP b126) and HapMap Project database (Release 27, Phase II + III). Linkage disequilibrium (LD) analysis of the common SNPs (minor allele frequency ≥ 0.05) revealed that all the SNPs except rs2293152 in *STAT3* gene with 5' flanking 2 kbps putative promoter region are sited in two blocks (*r*^2^ ≥ 0.8). We choose rs1053005 (located in the 3'-UTR) and rs957970 (an intronic SNP) as tagSNPs for the two blocks to study the association between the variants of *STAT3* and clinical phenotypes. Genomic DNA for analyses was isolated from blood samples. Genotyping of the polymorphisms was performed with the polymerase chain reaction-restriction fragment length polymorphism (PCR–RFLP) assay. Five percent of total samples were selected randomly for sequencing to confirm the accuracy of genotyping.

### 4.3. Statistical Analysis

Statistical Package for Social Sciences (version 16.0, SPSS Inc., Chicago, IL, USA) was used for Statistical analysis. Generalized linear model was used to determine associations between genotypes and quantitative clinical traits. Logistic regression model was used to estimate odds ratios (OR) of genotypes and 95% confidence interval (95% CI) for the risk of metabolic diseases adjusted for gender and age as appropriate. Frequency estimation of haplotypes and association analysis were performed using the haplo.stats package (version 1.6.3, http://cran.r-project.org/web/packages/haplo.stats/) for R (version 3.0.1, http://cran.cnr.berkeley.edu/). Chi-square test was used to assess the deviation of genotype distributions from Hardy-Weinberg equilibrium (HWE) and to compare genotypic and allelic frequencies between case and control groups. The significant level was set at 0.05.main text paragraph.

## 5. Conclusions

We identified two single nucleotide polymorphisms of *STAT3* associated with obesity or plasma triglyceride in a Chinese Han population. Our observations further confirm the involvement of STAT3 in regulation of energy homeostasis and lipid metabolism and indicate the potential for STAT3 to serve as a target for controlling the development of obesity and other metabolic disorders.

## Supplementary Files

Supplementary File 1
