# Supplementary material for: Association of STAT3 Common Variations with Obesity and Hypertriglyceridemia: Protective and Contributive Effects
Source: Int J Mol Sci. 2014 Jul 10;15(7):12258–69. doi: 10.3390/ijms150712258 (PMC4139842; doi:10.3390/ijms150712258)
Supplement: Supplementary File 1 [file ijms-15-12258-s001.pdf]

Supplementary Information

Figure 1. Linkage disequilibrium plot of HapMap SNPs in *STAT3* gene with 5' flanking 2 kb sequence. SNPs are displayed in reverse strand. Genotype data for SNPs in the region chr17:37718869..37795931 in Han Chinese in Beijing was downloaded from http://www.hapmap.org (Rel 27 Phase II + III, Feb09). SNPs with MAF ≥ 0.05 were analyzed to obtain the pairwise LD plot using the Tagger program in Haploview (version, 4.2). D' is indicated directly by both the brightness of red color and percentage. (**a**) TagSNP rs1053005 located in 3'-UTR captured the common SNPs in block 1; (**b**) TagSNP rs957970 located in an intron region captured the common SNPs in block 2; and (**c**) rs4796793 located in putative promoter region was in high linkage disequilibrium with rs957970 ($r^2 = 0.95$) and was predicted to generate a transcription factor binding site for FOXD1 by SNPInspector (version, 2.4).

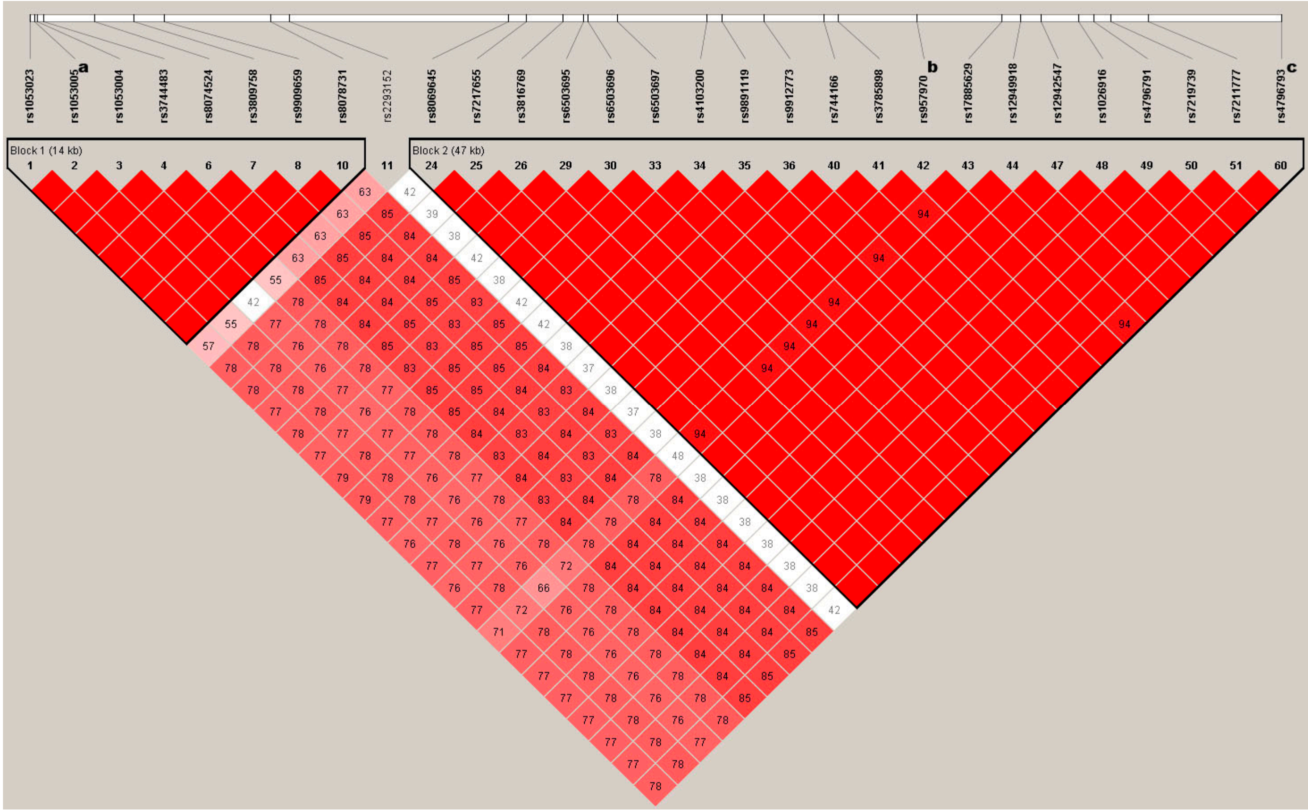

Figure 2. A>G variation of rs1053005 impairs the targeting of has-miR-1303 at STAT3 3' untranslated region. MicroRNA target prediction was performed using RNA22 program (version 1.0). STAT3 cDNA sequence is in accordance with ENST00000264657. * rs1053005 (A>G) is sited at the No. 4298 nucleotide of STAT3 cDNA. ":" represents uncomplementary base pairing.

```
                 4278                  4299
                  |                      *|
STAT3 cDNA   --> -GGGGGAGACGACCTTCTCTAAG
                  |:| :||||  || |||||||:
hsa-miR-1303 --> TCTCGTTCTGG-GGCAGAGATTT
                 |                      |
                 22                     1
```
